# The investigation of structural and vibrational properties and optical behavior of Ti-doped La_0.67_Ba_0.25_Ca_0.08_Mn_(1−*x*)_Ti_*x*_O_3_ (*x* = 0.00, 0.05 and 0.10) manganites

**DOI:** 10.1039/c9ra07407d

**Published:** 2019-12-19

**Authors:** Marwa Bourguiba, Zeineb Raddaoui, Moez Chafra, Jemai Dhahri

**Affiliations:** Laboratoire de Recherche en Mécanique Appliquée et Systèmes, Ecole Polytechnique de Tunisie, Université de Carthage La Marsa Tunisia; Faculté des Sciences de Tunis, Université de Tunis El Manar Tunis 2092 Tunisia; Laboratoire de La Matière Condensée et des Nanosciences, Département de Physique, Faculté des Sciences de Monastir, Université de Monastir 5019 Tunisia j.dhahri3000@gmail.com

## Abstract

The influence of Ti^4+^ ions incorporated into the B site on the structural, vibrational and optical properties of La_0.67_Ba_0.25_Ca_0.08_Mn_(1−*x*)_Ti_*x*_O_3_ (LBCM_(1−*x*)_T_*x*_), a polycrystalline compound prepared by a molten salt method, was discussed. The X-ray diffraction (XRD) studies confirmed that at room temperature these compounds crystallize in the rhombohedral phases of *R*3̄*c*. Rietveld refinement indicated that the octahedron (Mn/Ti)O_6_ underwent a slight deformation and the *θ*_(Mn/Ti)–O–(Mn/Ti)_ bond angles decreased with the increase in the Ti content. Furthermore, Raman spectra were recorded at room temperature for the LBCM_(1−*x*)_T_*x*_ ceramics to investigate the influence of incorporated Ti^4+^ ions in LBCM_(1−*x*)_T_*x*_. Moreover, we controlled the frequency and damping of the optic modes based on Ti incorporation. The infrared (IR) absorption spectrum (FTIR) analysis in the span of 420–750 cm^−1^ supports the XRD results. The diffuse reflectance data at room temperature verified that both transition levels (^5^E_g_ → ^5^T_5g_) and (^4^A_2g_ → ^4^T_2g_) correspond to the Mn^3+^ and Mn^4+^ ions. The optical band gap (*E*_g_) values decreased from 2.90 eV to 2.70 eV with the increase in the Ti^4+^ content, implying that our samples could be good candidates for some applications in luminescent devices, such as ultrafast optoelectronic devices. Moreover, the photoluminescence spectra (PL) features at room temperature decreased for all samples. CIE were estimated for all the concentrations of Ti^4+^ ions. The results indicated that are a shifts in the CIE values of the compounds.

## Abstract 2

The influence of Ti^4+^ ions incorporated into the B site on the structural, vibrational and optical properties of La_0.67_Ba_0.25_Ca_0.08_Mn_(1−*x*)_Ti_*x*_O_3_ (LBCM_(1−*x*)_T_*x*_), a polycrystalline compound prepared by a molten salt method, was discussed.

## Graphical abstract

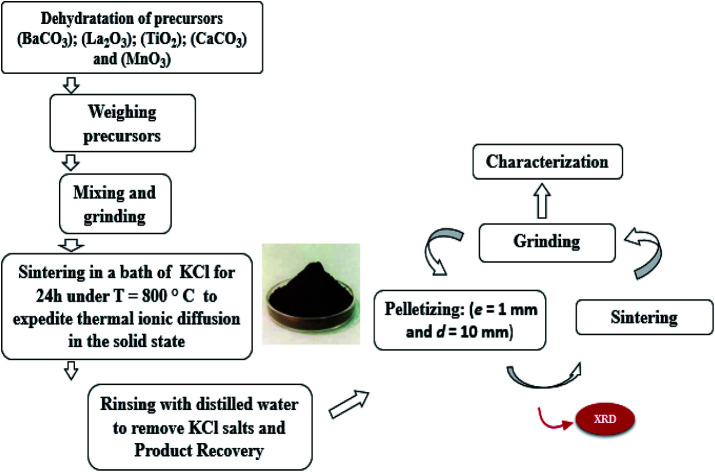

## 1. Introduction

Perovskite manganese oxides of Ln_1−*x*_A_*x*_MnO_3_ (A = Ca, Sr, and Ba) have been an important topic in scientific studies and potential technological applications due to their interesting physical properties, resulting from the orbital degree of freedom, lattice charge and spin coupling.^1,2^ These properties can be manipulated due to the flexibility of the lattice deformation,^3,4^ the filling of the one-electron level, the internal strain and its width. In fact, manganites, owing to their physical and chemical properties, have attracted much interest because of their novel magnetic, electronic and optical properties. Colossal magneto-resistance (CMR) has recently been a source of great interest for these manganites for their potential applications in magnetic storage systems, magnetic field sensors and spintronics.^5–10^ Recently, the exceptional properties of barium-doped lanthanum manganites (La_1−*x*_Ba_*x*_MnO_3_, LBM) have secured a prominent position in many industrial technologies, such as magnetic sensors.^11–14^ These manganites demonstrate a transition from metal behavior to insulator behavior, followed by a ferro–paramagnetic transition, *i.e.*, the Curie temperature (*T*_C_), which is characterized by high magnetic entropy.^15,16^ The substitution of bivalent cations for the rare-earth cations in manganites leads to converting twice as many Mn^3+^ into Mn^4+^ ions to donate information to the double exchange interactions, which are the origin of the ferromagnetic behavior.^17,18^

Moreover, the replacement of manganese by titanium ions has been the subject of diverse studies examining the magnetic, magneto-caloric and electrical behaviors of La_0.7_Sr_0.25_Na_0.05_Mn_(1−*x*)_Ti_*x*_O_3_,^19^ La_0.85_Ba_0.15_Mn_(1−*x*)_Ti_*x*_O_3_ (ref. 20) and La_0.57_Nd_0.1_Pb_0.33_Mn_1−*x*_Ti_*x*_O_3_.^21^ Kossi *et al.*^22^ investigated the impact of the incorporation of Ti^4+^ ions on the morphological and various electrical properties of a La_0.7_Sr_0.25_Na_0.05_Mn_0.9_Ti_0.1_O_3_ manganese perovskite. They confirmed that the lattice effects on the physical properties can be ignored since Mn^4+^ ions are replaced directly with Ti^4+^ ions. However, new insight into the physical properties with respect to the optical properties has not been established in manganites that either show insulator behavior (large band gap; typically, >4 eV) or metallic behavior (no band gap), which makes them less important for optical studies. Kumar *et al.*^23^ confirmed that the (La_0.6_Pr_0.4_)_0.65_Ca_0.35_MnO_3_ ceramic is a potential candidate for optical applications.

A detailed literature survey shows that not much work has been done on the vibrational and optical properties in the simultaneous substitution of Ba^2+^/Ca^2+^ ions and transition metal Ti^4+^ ions in manganese perovskites. For this reason, we report the effect of Ti^4+^ ions incorporated in the Mn sites on the structural, vibrational and optical properties of the polycrystalline LBCM_(1−*x*)_T_*x*_. This polycrystalline material is synthesized by the molten salt method.

## 2. Experimental details

### 2.1. Synthesis of La_0.67_Ba_0.25_Ca_0.08_Mn_(1−*x*)_Ti_*x*_O_3_ ceramics

**Fig. 1:**
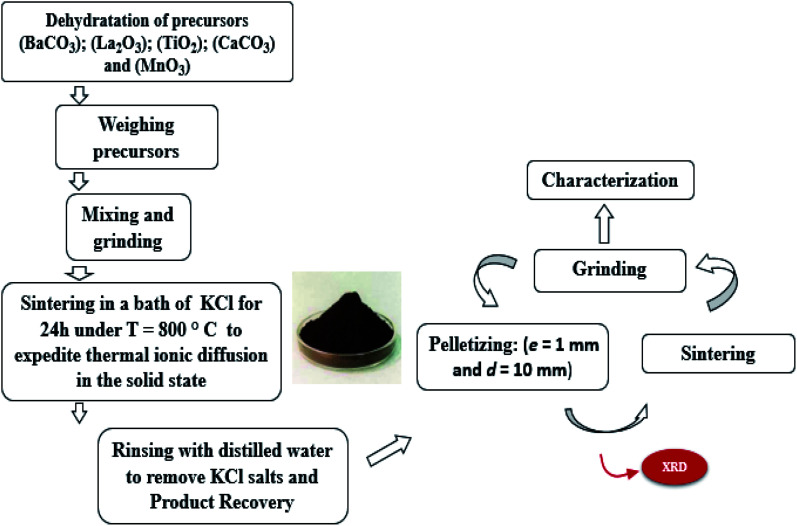
Flow-chart of LBCM_(1−*x*)_T_*x*_ prepared by a molten salt method.

The LBCM_(1−*x*)_T_*x*_ compounds were synthesized *via* a flux method. The metal precursors with high purity were La_2_O_3_, BaCO_3_, CaCO_3_ MnO_2_ and TiO_2_. Then, these precursors were mixed well for 2 h in an agate mortar and 2 h in alcohol. The resultant blends were heated at 800 °C for 24 h in an alumina melting-pot, and then cooled at room temperature. After the completion of the reaction, the rest were crushed and washed repeatedly with distilled water to remove the salts. After slowly drying at 100 °C in air, it was pressed into pellets (*e* = 1 mm and *d* = 10 mm) and sintered at 1000 °C for 24 h. For clarity, Fig. 1 shows the steps of the synthesis process.

### 2.2. Characterization

The crystalline phases of LBCM_(1−*x*)_T_*x*_ were checked by XRD using the “PANalytical X'Pert Pro” diffractometer (*λ*_Cu-Kα_ = 1.5406 Å). The spectra were noted in the 2*θ* region of 10°–60° with a pitch of 0.02°. The structural analysis was carried out using the FullProf program.^24,25^

The infrared spectra were measured by transmittance mode in the range 450 to 4000 cm^−1^ at room temperature on a PerkinElmer spectrum 100 spectrophotometer.

The Raman measurements were registered in the frequency region from 50 to 1000 cm^−1^ utilizing a LabRAM HR800. The compounds were excited using a 488 nm laser.

The UV-visible reflectance spectra were measured on a Shimadzu UV-3101PC spectrophotometer.

The photoluminescence (PL) spectra were collected at 300 K employing a iHR320 monochromator. The LBCM_(1−*x*)_T_*x*_ were excited using a 300 nm source.

## 3. Results and discussion

### 3.1. X-ray diffraction

**Fig. 2:**
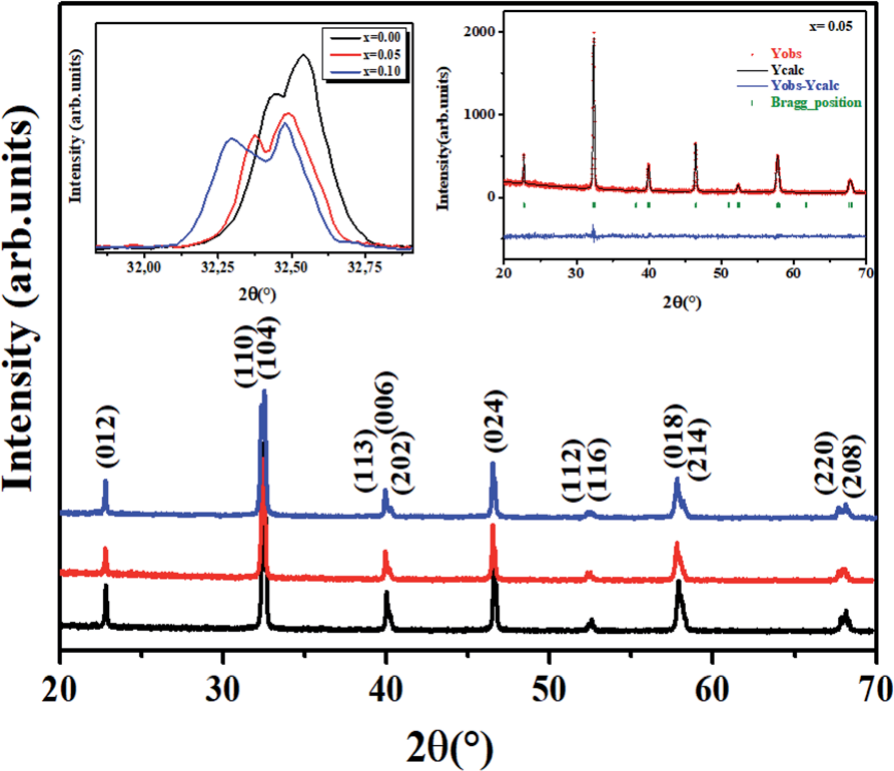
XRD pattern of LBCM_(1−*x*)_T_*x*_ ceramics with *x* = 0.00; 0.05 and 0.10. Open circles correspond to the X-ray diffraction data and the lines are theoretical fits to the observed X-ray data. Rietveld analysis results for the *x* = 0.05 sample. Vertical bars are the Bragg reflections for the *R*3̄*c* space group. The pattern difference between the observed data and the theoretical fit is shown at the bottom.

Fig. 2 shows the XRD data of all the LBCM_(1−*x*)_T_*x*_ samples at room temperature. These curves affirm the one phase nature of our compounds. A zoom-in of the most intense peak displays a shift to lower 2*θ* values (inset of Fig. 2), indicating that the unit cell volume rises with the increase in the Ti content. This increase affirmed the incorporation of Ti (*r*_Ti_^4+^ = 0.605 Å) at the Mn site and the ratio can explain the anisotropic contractions.^26,27^

The results of the structural refinement confirmed the rhombohedral phase *R*3̄*c* (no. 167), where (La/Ba/Ca) are at the 6a (0, 0, 1/4) position, (Mn/Ti) at 6b (0, 0, 0) and O at 18e (*x*, 0, 1/4).

The Rietveld refinement is depicted in the inset of Fig. 2 for *x* = 0.05 as an example. The performance of XRD data refinement is evaluated by the adjustment sign, such as the weighted pattern *R*_wp_, pattern *R*_p_, and the goodness of fit *χ*^2^. Good agreement was observed between the calculated and experimental XRD patterns. The results after fitting are illustrated in Table 1. In our case, it is worth remarking that the Ti^4+^ ionic radius (*r*_Ti_^4+^ = 0.605 A) is larger than that of Mn^4+^ (*r*_Mn_^4+^ = 0.53 A).

**Table 1:** Refined structure parameters at room temperature for LBCM_(1−*x*)_T_*x*_ (*x* = 0.00, 0.05 and 0.10)

La_0.67_Ba_0.25_Ca_0.08_Mn_(1−*x*)_Ti_*x*_O_3_	*x* = 0.00	*x* = 0.05	*x* = 0.10
**Cell parameters**			
*a* (Å)	5.530(7)	5.523(1)	5.515(2)
*c* (Å)	13.453(8)	13.550(3)	13.557(1)
*v* (Å^3^)	355.47(1)	357.648(2)	359.08(3)
*c*/*a*	2.435(8)	2.461(4)	2.450(2)

**Agreement factors**			
*R*_P_ (%)	9.54	10.7	6.56
*R*_WP_ (%)	12.2	13.6	8.55
*χ* ^2^	2.22	2.84	1.33

**Bond lengths and bond angles**			
*d*_Mn–O–Mn_ (Å)	1.957	1.970	1.970
*θ*_Mn−O_ (°)	169.64	165.20	163.93
*W* (u.a) × (10^−2^)	9.49	9.24	9.21

Therefore, the incorporation of Ti^4+^ into the Mn^4+^ site induces a deformation of the hexagonal phase by an elongation along both the *a* and *c* axes and, consequently, an increase of the cell volume.

The increase of the cell parameters is caused by the increase of the Mn–O bond length (〈*d*_Mn/Ti–O–Mn_〉).

**Fig. 3:**
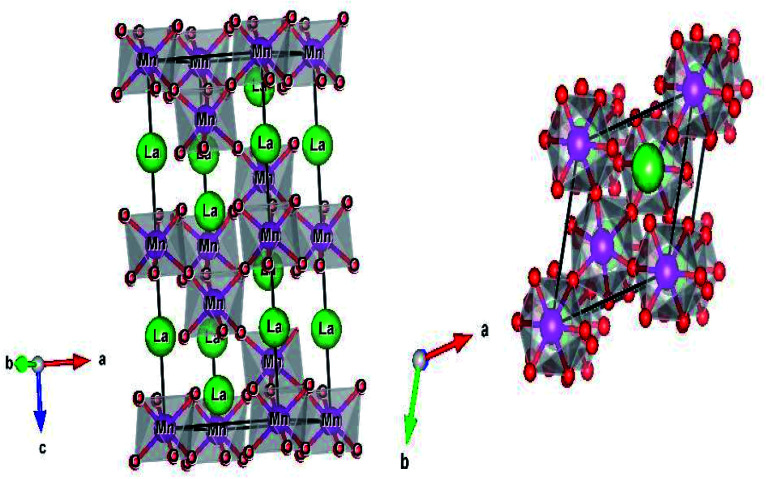
The crystal structure and the TiO_6_ octahedron for the LBCM_0.95_Ti_0.05_ sample.

The structure and the (Mn/Ti)O_6_ octahedron for the LBCM_0.95_T_0.05_ sample (as an example) were plotted by the “Diamond” program, which, based on the refined atomic positions, is depicted graphically in Fig. 3. From this figure, the lattice deformation can be seen. Therefore, lattice effects may impact the vibrational properties and optical behaviors in these compounds.

To better understand the influence of cation incorporation, Raman and infrared spectroscopy are useful techniques for examining the structure and identifying the functional groups in these compounds.

### 3.2 Raman spectroscopy investigation

Raman spectroscopy was carried out for all the samples in order to better understand the modification changes in the structure. In addition, the observed Raman spectra allow us to study the influence of the deformation introduced by the incorporation of Ti^4+^ ions at the B site and correlate between the structural details.

**Fig. 4:**
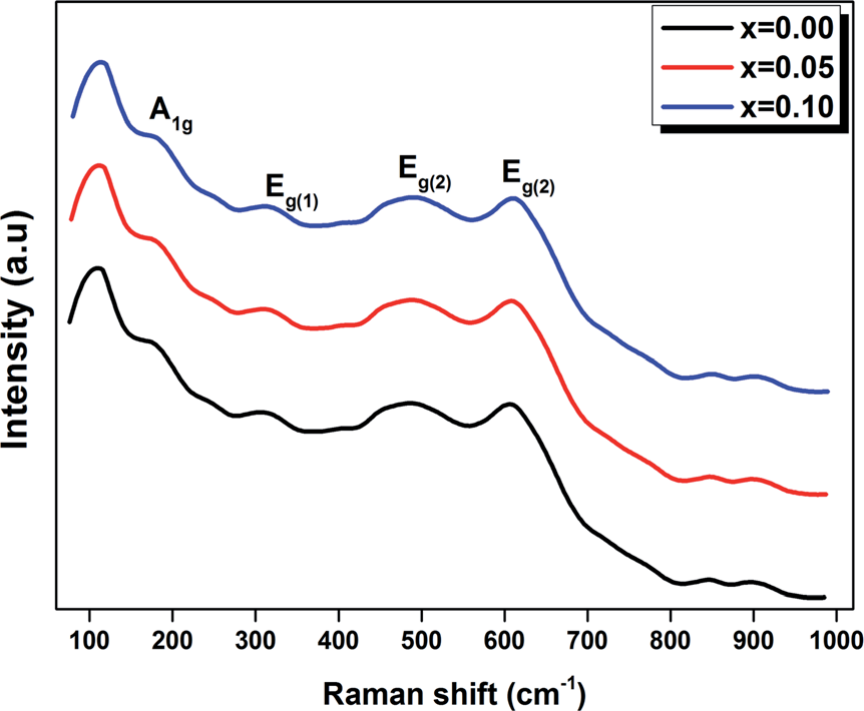
Raman spectra of LBCM_(1−*x*)_T_*x*_ ceramics with *x* = 0.00, 0.05 and 0.1 at room temperature.

Fig. 4 shows the Raman spectra in the range of 80–1000 cm^−1^ for the LBCM_(1−*x*)_T_*x*_ (*x* = 0.00, 0.05 and 0.10) manganite at room temperature.

We notice that the LBCM_(1−*x*)_T_*x*_ ceramics Raman spectra are very similar in position and profile to the data of the pure LBCM ceramic, which is characterized by the rhombohedral phase (*D*_3d_). These results are similar to other literature reports.^28–31^

In addition, we noticed the peak positions are shifted to higher frequencies and the intensity increases as the rate of Ti increases in our compounds. This behavior may be due to disorder in our compounds, which is followed by the Ti^4+^ substitution at the B site for the LBCM_(1−*x*)_T_*x*_ ceramic. Meanwhile, an important modification of the local vibrational dynamics generated by the structural distortion and slight modification in the local symmetry of the LBCM manganite (for *x* = 0.00) can be observed.

In our case, for the compound LBCM (*x* = 0.00), we observed five Raman modes at 110, 180, 309, 486 and 609 cm^−1^. This is in agreement with that reported for La_0.65_Eu_0.05_Sr_0.3−*x*_MnO_3_ by Bellouz *et al.*^32^ The band at low frequency occurring around 111 cm^−1^ is dominant since it corresponds to the distortions in the A-site cations (La/Ba/Ca). The peak at 180 cm^−1^ is assigned as A_1g_. The mode near 300–400 cm^−1^ corresponds to the E_g(1)_ mode, which are the bending, rotational and stretching vibrations of the MnO_6_ octahedra.^33–35^ We ascribe the E_g(2)_ mode (at 609 and 486 cm^−1^) to the Mn–O and O–Mn–O vibrations, respectively.

**Fig. 5:**
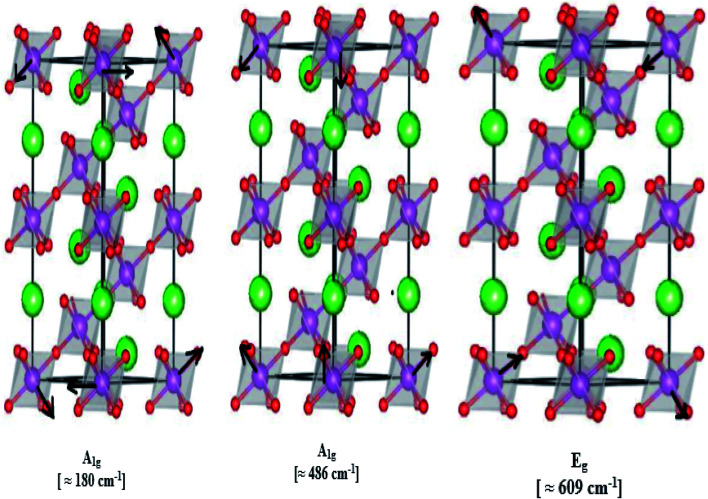
Representation of different A and E vibration in LBCM_0.95_T_*x*0.05_ ceramics, as an example.

Fig. 5 illustrates the ionic pattern associated with the A and E vibration in a rhombohedral of LBCM_0.95_T_0.05_, as an example.

For LBCM_(1−*x*)_T_*x*_ (*x* = 0.05 and 0.10) compounds, they are similar to the compound where *x* = 0.00, *i.e.* there are no new peaks during incorporation. The Ti ions do not substitute exactly for Mn ions, and a displacement of the smaller Ti with respect to the Mn site is responsible for a modified local symmetry, which can result in the activation of phonons out of the Brillouin zone center.

In addition, the frequency and damping of the three modes 110, 490 and 605 cm^−1^ for the LBCM_(1−*x*)_T_*x*_ samples is listed in Table 2. We confirm that the band wavenumber shifts slightly to a higher value. This shift, due to the incorporation of partial Ti ions, leads to the deformation of the octahedral (MnO_6_) of our perovskite, and so allows for a modification in the Mn–O–Mn bond angle. These vibration modes in manganites arise from the Jahn–Teller (J–T) distortions. Furthermore, we underscore that the (E_g_) mode (≈609 cm^−1^) originates from the symmetric stretching vibration of oxygen in MnO_6_ octahedra.

**Table 2:** Frequency and damping of modes in LBCM_(1−*x*)_T_*x*_ (*x* = 0.00, 0.05 and 0.10) at room temperature

Compounds	La_0.76_Ba_0.25_Ca_0.08_Mn_(1−*x*)_Ti_*x*_O_3_					
	0.00		0.05		0.10	
Phonon	Frequency (cm^−1^)	Damping (cm^−1^)	Frequency (cm^−1^)	Damping (cm^−1^)	Frequency (cm^−1^)	Damping (cm^−1^)
Vibration of A site	110.8	5.19	115	4.815	115	5.528
E_g(1)_	490	3.7	493	3.8	500	4.19
E_g(2)_	605	8.9	606	9.02	607	9.05

So, the damping of the phonons is also much more important in the LBCM_0.9_T_0.10_ sample than in pure LBCM and the LBCM_0.95_T_0.05_ sample. The contrast is most obvious with the highest frequency mode E_g(2)_, whose damping and frequency are unaffected.

This can be related to the fact that as the (Ca, Ba) ion radius is notably smaller than that of the substituted La ion, it can participate easily in A-site and A_1g_ motions. On the contrary, the E_g(2)_ mode is controlled by the introduction of Ti ions into the B site of LaMnO_3_ ceramics.

**Fig. 6:**
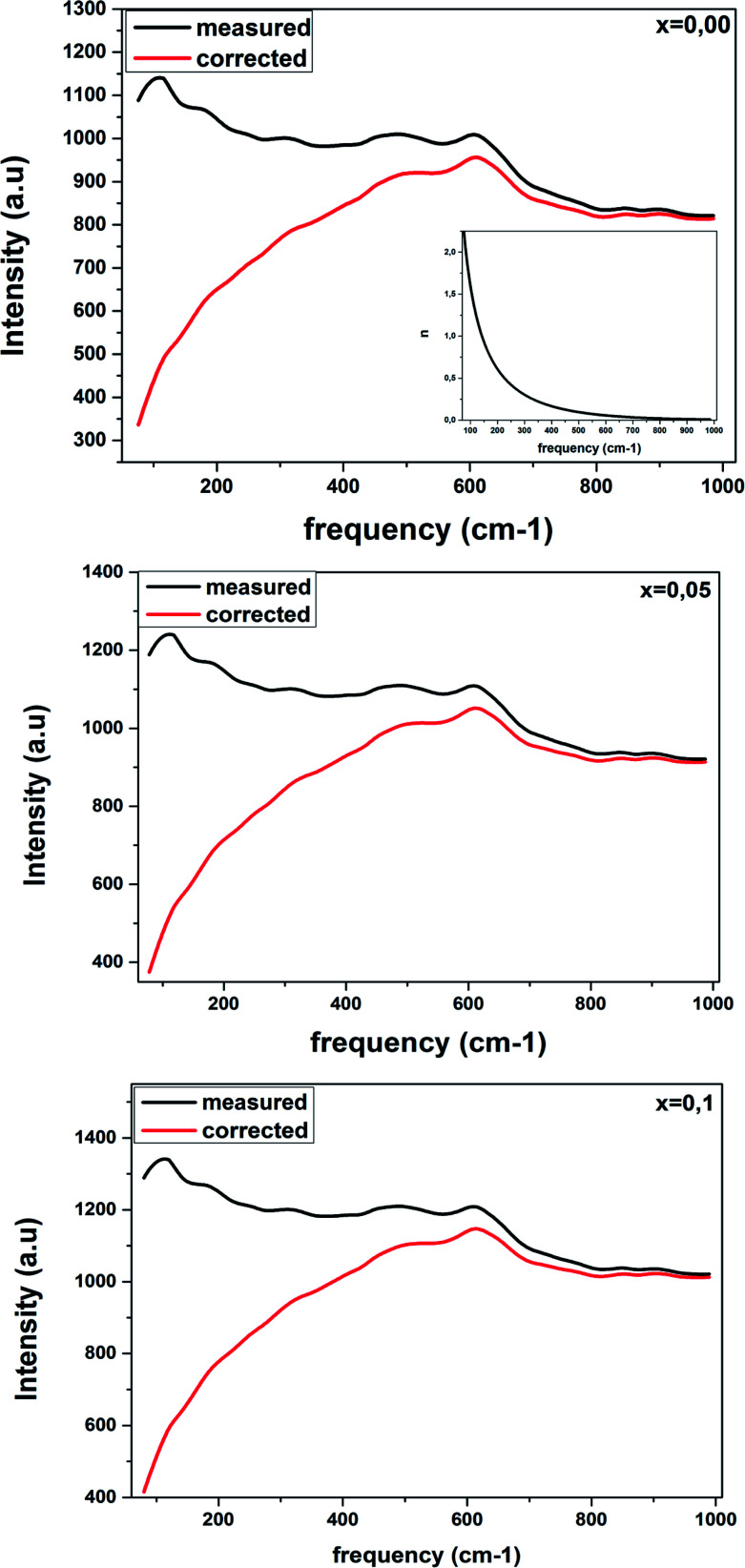
Corrected Raman spectra of LBCM_(1−*x*)_T_*x*_ ceramics are obtained after dividing the measured data by the Bose–Einstein population factor (reported in the inset).

Fig. 6 shows the Raman response that has been rectified by the population factor *n*(*ω*) = (e^*ℏω*/*kT*^ − 1)^−1^. It allows us to eliminate supplementary bands as artifacts within the fitting process. We remark that the factor *n*(*ω*) + 1 is related to first-order Stokes scattering.

At low frequencies, we notice a disappearance in the modes when comparing between the original spectra and after dividing the Raman response by the factor *n*(*ω*) + 1. This results have influenced on the optical response at room temperature.

### 3.3. Fourier transform infrared spectroscopy

**Fig. 7:**
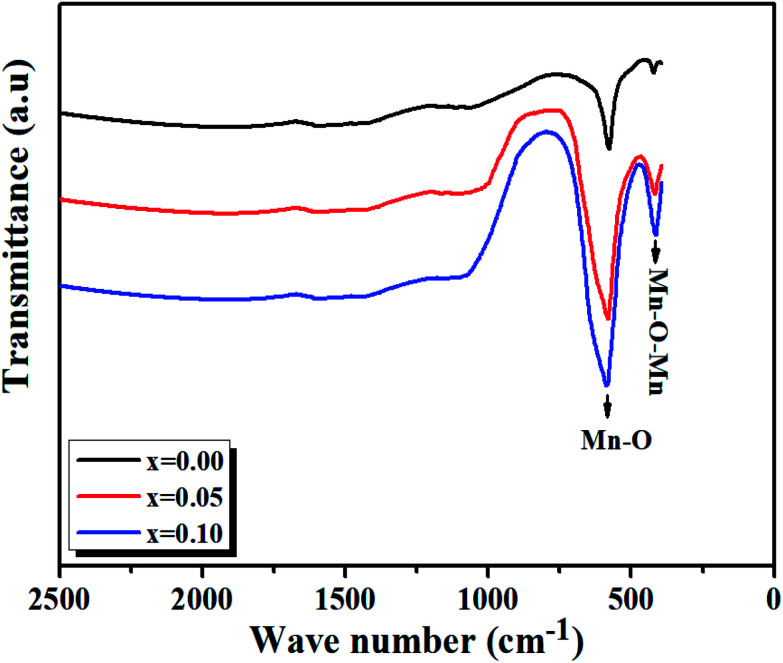
FTIR spectra of LBCM_(1−*x*)_T_*x*_ (*x* = 0.00, *x* = 0.05 and *x* = 0.10) ceramics at room temperature.

At room temperature, the Fourier transform infrared absorption spectra (FTIR) are illustrated in Fig. 7. We notice our samples exhibit mainly two transmission bands in the range of 420–750 cm^−1^. The first band is probably due to the folding mode, *ν*_b_, at about 420 cm^−1^, which is susceptible to the bonding angle Mn/Ti–O–Mn/Ti (〈*θ*_Mn/Ti–O–Mn/Ti_〉).

The second band is due to the stretching mode, *ν*_S_, at about 600 cm^−1^, implying that the internal movement of the Mn^4+^ ion is opposed to the (Mn/Ti)O_6_ octahedron resulting from the Jahn–Teller (J–T) effect.^30,31^

The transmission wave number (*ν*) of the Mn/Ti–O bond vibration is given by Hooke's relation:1
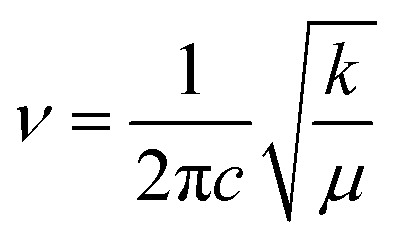
where *k* is the average force constant of the Mn/Ti–O bond (〈*d*_Mn/Ti–O–Mn_〉), *c* is the velocity of light, and *μ* is the effective mass of the Mn/Ti–O bond expressed by the following relation:2
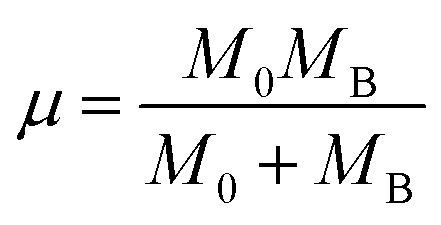
where *M*_0_ is the atomic weight of O, and *M*_B_ is the atomic mass of the B site ions given by the relation:

3 *M*_B_ = (1 − *x*)*M*_Mn_ + *xM*_Ti_

where *M*_Ti_ and *M*_Mn_ are the atomic weights of Ti and Mn, respectively.

The force constant can be connected to the average Mn/Ti–O bond length (*r*) by the following expression:4
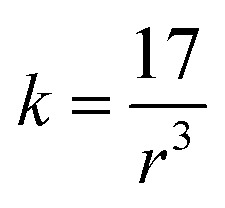


Based on eqn (1)–(4), the effective mass value, Mn/Ti–O bond lengths and the force constant for all compounds were determined from the FTIR spectra and are summarized in Table 3. We can remark that the calculated Mn/Ti–O bond lengths from the FTIR spectra are very close to the results obtained from the Rietveld refinement.^36–38^

**Table 3:** IR bands, effective mass, force constant and B–O bond length values for LBCM_(1−*x*)_T_*x*_ (*x* = 0.00, 0.05 and 0.10) samples

Compositions	Wavenumber (cm^−1^)	Effective mass (10^−26^ kg)	Force constant (N cm^−1^)	Bond length (B–O) (from FTIR) (Å)	Bond length (B–O) (from Rietveld) (Å)
*x* = 0.0	578.59	2.0576	2.4474	1.900	1.957
*x* = 0.05	579	2.0546	2.44729	1.9080	1.970
*x* = 0.10	582	2.0516	2.469	1.9094	1.9703

From Fig. 7, it is observed that a remarkable change in the FTIR occurs, in which the transmission bands associated with the Mn/Ti–O stretching vibration shift from 577.57 cm^−1^ to 585.49 cm^−1^ with the increasing value of *x* and increase in Mn/Ti–O bond lengths (*i.e.*, decrease in force constant (*k*)).

So, the results establish by FTIR, whither the frequency shift is expected by the deformation structure (see Fig. 4), a more disordered structure will created by a shift of the E_g_ mode in Raman spectra too.

### 3.4 Optical properties

#### 3.4.1 UV-visible diffuse reflectance

**Fig. 8:**
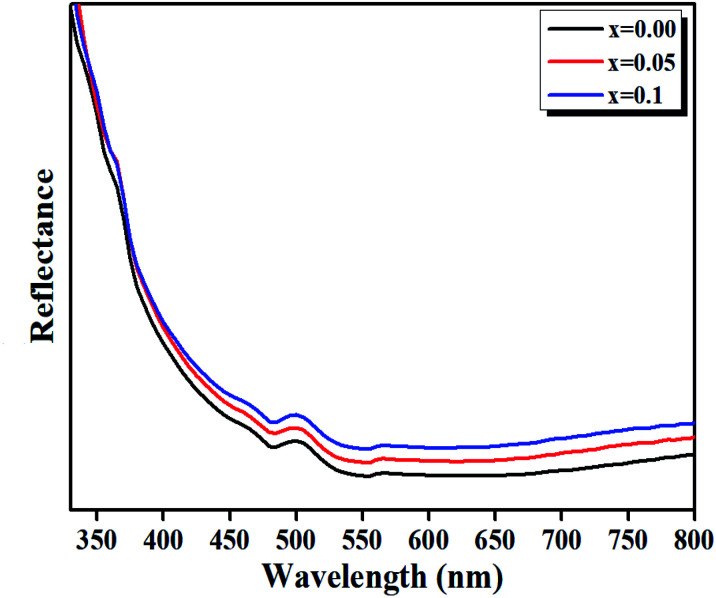
Diffuse reflectance spectra of LBCM_(1−*x*)_T_*x*_ (*x* = 0.00, *x* = 0.05 and *x* = 0.10) ceramics in the IR-vis region at room temperature.

At room temperature, the UV-visible diffuse reflectance data of the LBCM_(1−*x*)_T_*x*_ (*x* = 0.00, 0.05 and 0.10) ceramic samples are represented in Fig. 8.

The bands at 500 nm for these compounds are ascribed to d–d transitions of (^5^E_g_ → ^5^T_5g_) and (^4^A_2g_ → ^4^T_2g_) for the Mn^3+^ and Mn^4+^ ions, respectively.^39^

From the UV-visible reflectance data, the optical band gap energy (*E*_g_) was determined by the Kubelka and Munk method.^40^ This method is usually employed to study the diffuse reflectance measurement acquired from faintly absorbing compounds. In our case, the Kubelka–Munk equation for any wavelength is described by:5
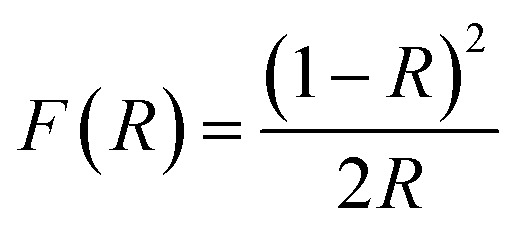
where *R* is the absolute reflectance of the compounds and *F*(*R*) is the so-called Kubelka–Munk function.

The absorption coefficient (*α*) can be given by the values of *F*(*R*):^41,42^6
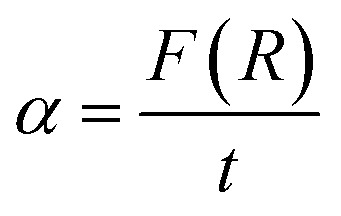
where *t* is the thickness of the LBCM_(1−*x*)_T_*x*_ compound which approximately equals 1 mm.

The optical band gap (*E*_g_) values of our ceramics were then calculated by the following equation:^43^

7 *αhν* = *A*(*hν* − *E*_g_)^*n*^

8 *F*(*R*)*hν* = *A*(*hν* − *E*_g_)^*n*^

where *hν* is the photon energy, *A* is an energy independent constant and *n* is a constant associated with the different types of electronic transitions (*n* = 1/2 for the direct allowed, *n* = 2 for the indirect allowed, *n* = 3/2 for the direct forbidden and *n* = 3 for the indirect forbidden).

However, it is likely that only direct transitions will occur in the perovskite.

Consequently, eqn (8) becomes:

9 [*F*(*R*)*hν*]^1/2^ = *A*(*hν* − *E*_g_)

We have plotted the variation of [*F*(*R*)*hν*]^1/2^*versus* (*hν*) for our compounds. The values of the indirect optical band gap (*E*_g_) for the LBCM_(1−*x*)_T_*x*_ (*x* = 0.00, 0.05 and 0.10) were obtained, as represented in Fig. 8, by adjustment of the linear part of these plots at [*F*(*R*)*hν*]^1/2^ = 0. The *E*_g_ values are presented in Table 4.

**Table 4:** Band gap energy (*E*_g_) and Urbach energy (*E*_u_) for LBCM_(1−*x*)_T_*x*_ ceramics with *x* = 0.00; 0.05 and 0.10

La_0.76_Ba_0.25_Ca_0.08_ Mn_(1−*x*)_Ti_*x*_O_3_	*E*_g_ (eV)	*E*_u_ (eV)
*x* = 0.00	2.90	1.25
*x* = 0.05	2.80	1.3
*x* = 0.10	2.70	1.35

The values of the optical band gap are 2.90, 2.80 and 2.70 eV for *x* = 0.00, 0.05 and 0.10 respectively.

We notice that the *E*_g_ values decreased with the increase of Ti^4+^ rate in the LBCM_(1−*x*)_T_*x*_O_3_ ceramic. This shift of the *E*_g_ in the samples may be attributed to the modification in the Mn/Ti–O bond length and Mn/Ti–O–Mn/Ti bond angle, since cation incorporation plays a critical role in changing the one-electron bandwidth (*W*) and the optical band gap of the LBCM_(1−*x*)_T_*x*_O_3_ manganite ceramic, which is consistent with our XRD results.

The one electron bandwidth (*W*) relies on both the bond angle and bond length, as shown in this formula, *W*∞cos *ω*/*d*_Mn/Ti–O_^3.5^, where *ω* is 1/2[π − (Mn/Ti–O–Mn/Ti)] and *d*_(Mn/Ti–O)_ is the Mn/Ti–O bond length^44^ (see in Table 1).

Further, *E*_g_ is connected with *W* as follows: *E*_g_ = *Δ* − *W*, where *Δ* is the charge-transfer energy.^45^

In our case, all these structural changes are explained in a clean decrease of the one electron bandwidth (*W*). So, the substitution of Ti into Mn in the A site results in a decrease in the optical band gap.

In addition, the width of the defect bands existing in the optical band gap correspond to the Urbach energy, *E*_u_.^46^ It can be described by the equation:10
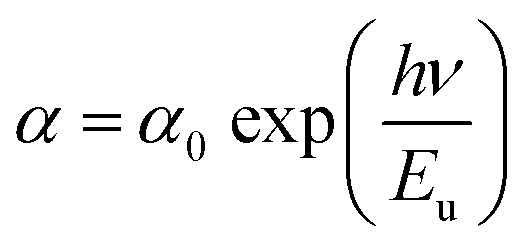


**Fig. 9:**
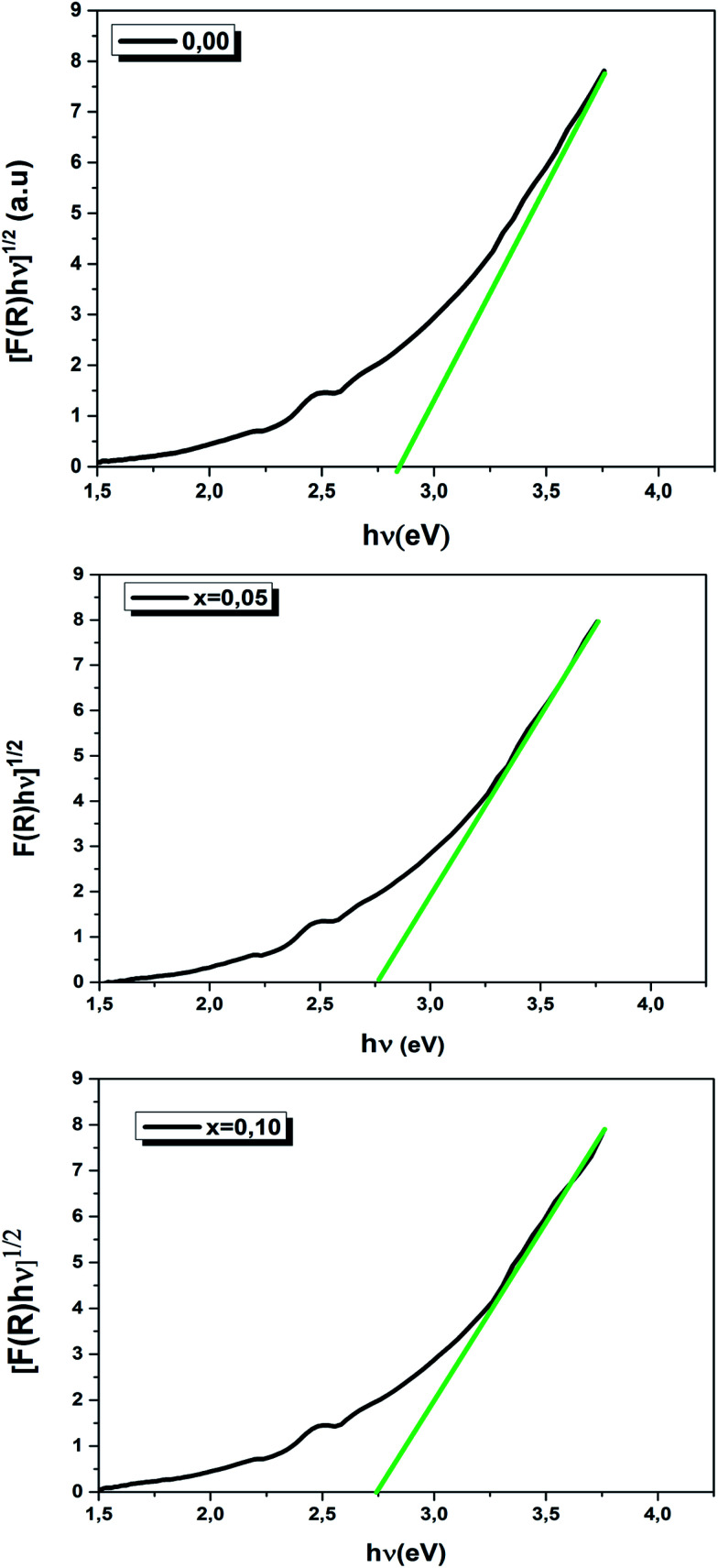
Energy band gap as a function of LBCM_(1−*x*)_T_*x*_ (*x* = 0.00, *x* = 0.05 and *x* = 0.10).

The Urbach energy, *E*_u_, was determined by plotting ln (*α*) *vs. hv* (Fig. 9).

The *E*_u_ values are also regrouped in Table 4. A slight increase in the Urbach energy values is observed with the addition of Ti^4+^ ions.

**Fig. 10:**
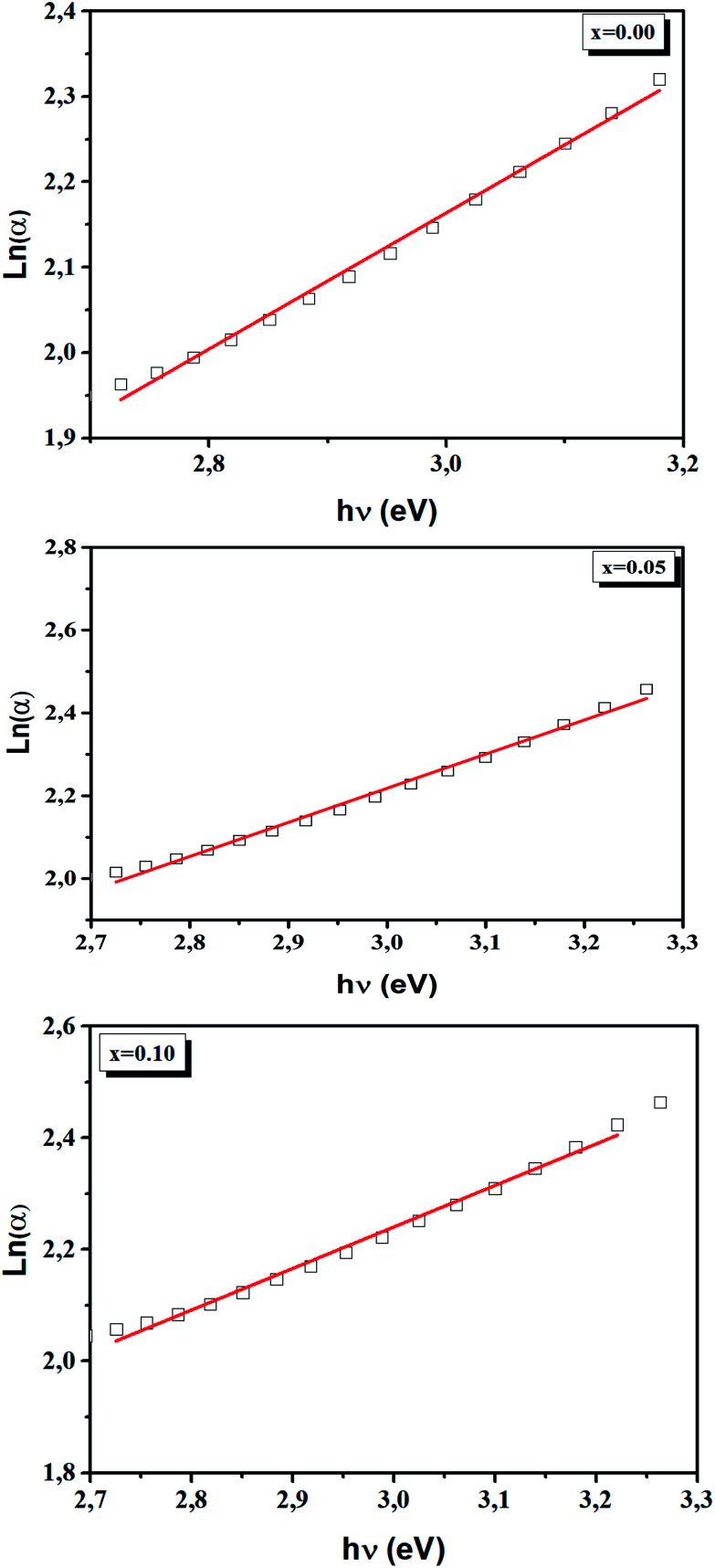
Plot ln (*α*) *versus* photon energy (*hv*) for LBCM_(1−*x*)_T_*x*_ (*x* = 0.00, *x* = 0.05 and *x* = 0.10).

For our sample, the LBCM_(1−*x*)_T_*x*_ ceramics, the optical band gaps span over 2.72–2.89 eV in the UV range, allowing for carrier excitation with femtosecond laser pulses, which makes these ceramics viable for applications in ultrafast optoelectronic devices (Fig. 10).^47^

#### 3.4.2 Photoluminescence study

**Fig. 11:**
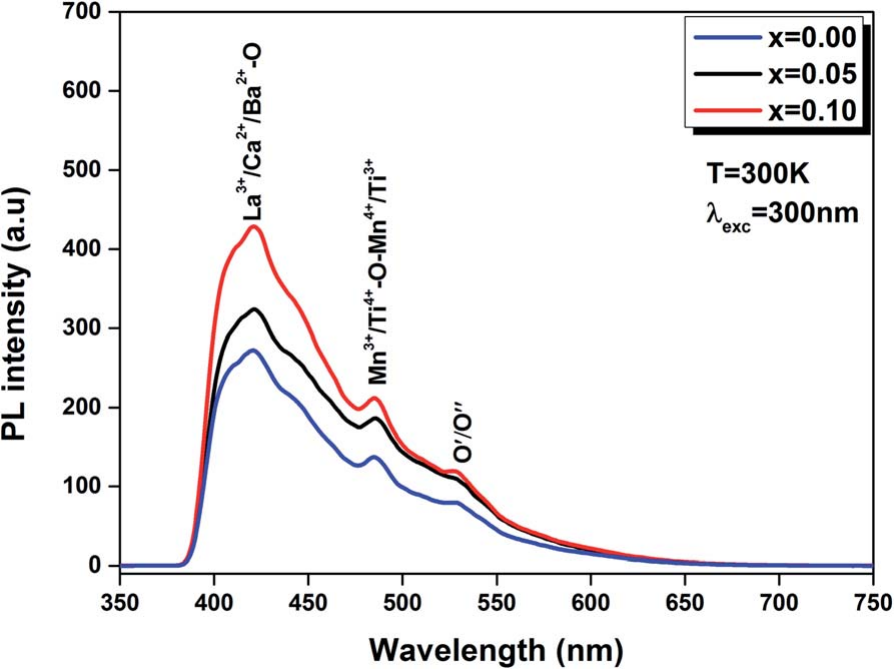
PL spectra of the LBCM_(1−*x*)_T_*x*_ (*x* = 0.00, *x* = 0.05 and *x* = 0.10) ceramics excited at 300 nm at room temperature.

The emission response of LBCM_(1−*x*)_T_*x*_O_3_ (*x* = 0.00, 0.05 and 0.10) at room temperature is illustrated in Fig. 11. We notice that the profiles of the PL spectra are similar to the pure sample.

Furthermore, we note that when titanium is introduced in the LBCM_(1−*x*)_T_*x*_ ceramic, the PL response intensity increases. This is demonstrated by the increase of defects in our compound.

The existence of (Ca^2+^, Ba^2+^) for La^3+^ in the A site and Ti^4+^ for Mn^3+^ in the B site of the LaMnO_3_ lattice results in a distortion of the lattice. In our case, the Ti^4+^ ions approach each other, which reinforces their interactions and results in quenched emission by non-radiative pathways (*i.e.* lattice vibrations). The experimental results show that photoluminescence is connected to the structural disorder and deformation.

The same result was reported by Zhu *et al.*^48^ for La_0.67_Ca_0.33_MnO_3_ compounds and Chen *et al.*^49^ for La_0.825_Sr_0.175_MnO_3_ compounds.

In addition, this comportment can be produced by oxygen vacancies for random common “perovskites” and/or disorder coupled to the “tilt” of a [Mn/TiO_6_]–[Mn/TiO_6_] complex cluster.

**Fig. 12:**
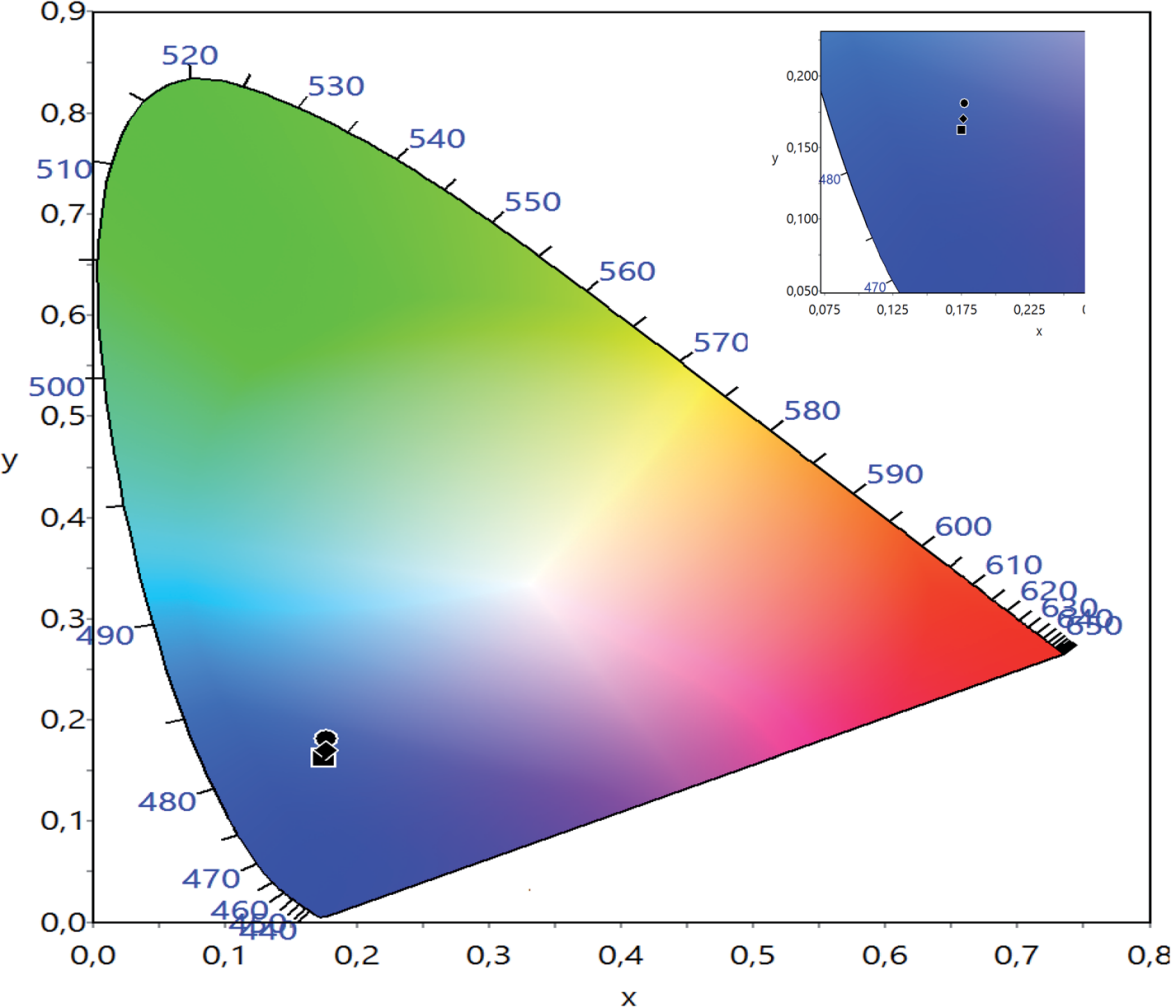
CIE chromatic diagram showing the influence of thermal treatment on the chromatic coordinates of LBCM_(1−*x*)_T_*x*_ (*x* = 0.00, *x* = 0.05 and *x* = 0.10).

The CIE chromaticity of LBCM_(1−*x*)_T_*x*_ (*x* = 0.00, 0.05 and 0.10) is illustrated in Fig. 12. The CIE diagram shows that the estimated coordinates are associated in the blue region and tuned towards the pure blue region. The calculated values of the CIE coordinates of the LBCM_(1−*x*)_T_*x*_ ceramic are tabulated in Table 5. The CIE coordinates vary from (0.1752, 0.1626) to (0.1773, 0.1688) upon the incorporation of Ti ions in the LBCM_(1−*x*)_T_*x*_ ceramic.

**Table 5:** The values of CIE (*x*, y) co-ordinates of LBCM_(1−*x*)_T_*x*_ (*x* = 0.00; 0.05 and 0.10)

Composition	CIE	
	*x*	*y*
*x* = 0.00	0.1752	0.1626
*x* = 0.05	0.1772	0.1812
*x* = 0.10	0.1773	0.1688

Increasing the Ti^4+^ ion rate in the host lattice led to significant modifications of the optical behavior, which can be attributed to the structural deformation. However, our investigation of the structural, vibrational and optical properties of the polycrystalline LBCM_(1−*x*)_T_*x*_ demonstrate that their optical sensitivity places them as good candidates for many practical applications in luminescent devices.

## 4. Conclusion

Polycrystalline LBCM_(1−*x*)_T_*x*_ (*x* = 0.00, 0.05 and 0.10) ceramics were synthesized by the molten salt process. The XRD analysis indicated that the ceramic possesses a rhombohedral phase structure *R*3̄*c*. The XRD analysis, along with the Raman and FTIR measurements, verify the incorporation of Ti into the Mn-site and the ensuing chemical disorder in these compounds. The optical band gap (*E*_g_) of our samples, as measured by UV-visible reflectance, decreases from 2.90 eV to 2.70 eV with the increase in the Ti content. The photoluminescence spectra (PL) features at room temperature are smaller for all samples. This behavior is explained by oxygen vacancies. The CIE coordinate varies from (0.1752, 0.1626) to (0.1773, 0.1688) upon the incorporation of Ti ions at Mn ion sites in the LBCM_(1−*x*)_T_*x*_ ceramic.

The investigation of the structural, vibrational and optical properties for the LBCM_(1−*x*)_T_*x*_ manganites shows that our compounds can be a good candidate for applications in luminescent devices, such as ultrafast optoelectronics.

## Conflicts of interest

There are no conflicts to declare.

## Supplementary Material

## References

[cit1] Dagotto E., Hotta T., Moreo A. (2001). Phys. Rep..

[cit2] Gorkov L. P., Kresin V. Z. (2004). Phys. Rep..

[cit3] Millis A. J., Littlewood P. B., Shraiman B. I. (1995). Phys. Rev..

[cit4] Millis A. J., Shraiman B. I., Mueller R. (1996). Phys. Rev. Lett..

[cit5] Bridges F., Booth C. H., Kwei G. H., Neumeir J. J., Swatzky G. A. (2000). Phys. Rev. B.

[cit6] Awana V. P. S., Tripathi R., Kumar N., Kishan H., Bhalla G. L., Zeng R., Chandra L. S. S., Ganesan V., Habermeier H. U. (2010). J. Appl. Phys..

[cit7] Haghiri-Gosnet A. M., Renard J. P. (2003). J. Phys. D: Appl. Phys..

[cit8] Samanta T., Das I., Banerjee S. (2007). Appl. Phys. Lett..

[cit9] Moreo A., Yunoki S., Dagotto E. (1999). Science.

[cit10] Mahato R. N., Sethupathi K., Sankaranarayanan V. (2010). J. Appl. Phys..

[cit11] Zeydi I., Zaidi A., Dhahri J., Hlil E. K. (2018). J. Magn. Magn. Mater..

[cit12] Kar M., Ravi S. (2002). Pramana.

[cit13] Srivastava S. K., Kar M., Ravi S. (2008). Mater. Sci. Eng., B.

[cit14] Srivastava S. K., Ravi S. (2009). J. Magn. Magn. Mater..

[cit15] Dhahri Ah., Dhahri E., Hlil E. K. (2019). RSC Adv..

[cit16] Laouyenne M. R., Baazaoui M., Farah Kh., Hlil E. K., Oumezzine M. (2018). J. Magn. Magn. Mater..

[cit17] Zner C. (1951). Phys. Rev..

[cit18] Kar M., Ravi S. (2004). Mater. Sci. Eng., B.

[cit19] Kossi S. E. L., Ghodhbane S., Mnefgui S., Dhahri J., Hlil E. K. (2015). J. Magn. Magn. Mater..

[cit20] Nadhira H., Kurniawan B., Arifni H., Ahmiatri S. (2013). Conference Proceedings.

[cit21] Dhahri A., Dhahri J., Hlil E. K., Dhahri E. (2012). J. Supercond. Novel Magn..

[cit22] Kossi S. E. L., Rhouma F. I. H., Dhahri J., Khirouni K. (2014). Phys. B.

[cit23] Kumar S., Dwivedi G. D., Kumar S., Mathur R. B., Saxena U., Ghosh A. K., Joshi A. G., Yang H. D., Chatterjee S. (2015). Dalton Trans..

[cit24] Rietveld H. M. (1969). J. Appl. Crystallogr..

[cit25] Roisnel T. and Rodriguez-Carvajal J., Computer Program FULLPROF, LLB-LCSIM, May, 2003

[cit26] Zhang S.-y., Zhao P., Cheng Z.-h., Li R.-w., Sun J.-r., Zhang H.-w., Shen B.-g. (2001). Phys. Rev. B.

[cit27] Vertruyen B., Hébert S., Maignan A., Martin C., Hervieu M., Raveau B. (2004). J. Magn. Magn. Mater..

[cit28] Fedorov I., Lorenzana J., Dore P., De Marzi G., Maselli P., Calvani P. (1999). Phys. Rev. B.

[cit29] Yahia M., Batis H. (2003). J. Inorg. Chem..

[cit30] Keshri (Shaw S., Joshi L., Rout S. K. (2009). J. Alloys Compd..

[cit31] Kolat V. S., Gencer H., Gunes M., Atalay S. (2007). Mat. science. engineering, B.

[cit32] Bellouz R., Oumezzine M., Dinia A., Schmerber G., Hlil El-K., Oumezzine M. (2015). RSC Adv..

[cit33] Martín-Carrón L., de Andrés A., Martínez-Lope M. J., Casais M. T., Alonso J. A. (2002). Phys. Rev. B.

[cit34] Podobedov V. B., Romero D. B., Weber A., Rice J. P., Schreekala R., Rajeswari M., Ramesh R., Venkatesan T., Drew H. D. (1998). Appl. Phys. Lett..

[cit35] Oumezzine M., Hassayoun O., Bellouz R., Bezerra Sales H., Hlil E. K. (2017). J. Alloys Compd..

[cit36] Bellouz R., Oumezzine Ma., Hlil E., Oumezzine M. (2016). Mater. Res. Bull..

[cit37] Varshney D., Shaikh M. W. (2014). J. Alloys Compd..

[cit38] Dodiya N., Varshney D. (2013). J. Mol. Struct..

[cit39] Jin Y., Hu Y., Wu H., Duan H., Chen L., Fu Y., Ju G., Mu Z., He M. (2016). Chem. Eng. J..

[cit40] Kubelka P., Munk F. Z. (1931). Tech. Phys..

[cit41] Yakuphanoglu F., Mehrotra R., Gupta A., Muñoz M. (2009). J. Appl. Polym. Sci..

[cit42] Yassitepe E., Khalifa Z., Jaffari G. H., Chou C.-S., Zulfiqar S., Sarwar M. I. (2010). et al.. Powder Technol..

[cit43] Le T. L., Guillemet-Fritsch S., Dufour P., Tenailleau C. (2016). Thin Solid Films.

[cit44] Radaelli P. G., Iannone G., Marezio M., Hwang H. Y., Cheong S. W., Jorgensen J. D., Argyriou D. N. (1997). Phys. Rev. B.

[cit45] Medarde M., Mesot J., Lacorre P., Rosenkranz S., Fischer P., Gobrecht K. (1995). Phys. Rev. B.

[cit46] Urbach F. (1953). Phys. Rev..

[cit47] Kumar P., Kar M. (2014). J. Alloys Compd..

[cit48] Zhu W. L., Ma Y. Q., Wu M. Z., Li H., Cao S., Yin W. J., Yang K., Zheng G. H., Sun Z. Q. (2009). Mater. Res. Bull..

[cit49] Chen F., Liu H. W., Wang K. F., Yu H., Dong S., Chen X. Y., Jiang X. P., Ren Z. F., Liu J.-M. (2005). J. Phys.: Condens. Matter.

